# Effects of genotype-by-environment and analysis of potential management interaction on vase life in cut lisianthus

**DOI:** 10.3389/fpls.2025.1578100

**Published:** 2025-05-15

**Authors:** Steven Kim, Seong Heo

**Affiliations:** ^1^ Department of Mathematics and Statistics, California State University, Seaside, CA, United States; ^2^ Department of Horticulture, Kongju National University, Yesan, Republic of Korea

**Keywords:** AMMI model, G × E, linear mixed-effects model, lisianthus, salicylic acid, vase life

## Abstract

This research aimed to analyze how different genotypic and environmental conditions, along with salicylic acid (SA) treatment (management), influence the longevity of cut lisianthus flowers during post-harvest period. Four genotypes (“Arena Green”, “Blue Picote”, “Corelli Pink”, or “Kroma White”), four environments (hydroponic or soil cultivation with SA treatment during vegetative or reproductive period), and four levels of managements (SA concentration at 0, 0.1, 0.3, or 0.5 mM) were analyzed using the additive main effects and multiplicative interaction model (also known as AMMI model) and linear mixed-effects regression models. The biplot and linear mixed-effects regression analysis showed that hydroponic cultivation with SA treatment during the reproductive period was the most effective environment for prolonging the vase life. It appeared that higher SA concentrations increased the vase life on average, but the effect of SA management depended on genotype and environment. In addition, the regression analysis revealed that dry weight and the interaction between petal number and petal size, among all measured vegetative and reproductive variables, were significantly related to the vase life. The regression lines indicated that the expected vase life increases with respect to the petal size when the petal number is low, but decreases when the petal number is high. In conclusion, genotype-specific cultivation and management is needed for enhancing the vase life of cut lisianthus flowers, and balance between petal size and petal number is also crucial. The findings suggest that an optimal strategy for improving the vase life depends on the environment, management, and genotype.

## 1 Introduction

Lisianthus (*Eustoma grandiflorum*) is an ornamental plant of the Gentianaceae family. Recently, the market has demonstrated robust growth for cut flowers, with sales now exceeding four billion dollars worldwide (Sukpitak et al., 2024), and there has been a surge in sales of lisianthus. It is dubbed the “next rose” due to its rose-like flower size, long stems, various flower coloration and morphology, and extended vase life (Liang et al., 2022). The vase life of cut flowers is defined as the durability time until they lose visual quality while they remain in the vase solution. The vase life is highly variable depending on the genotype (cultivar) and environment (cultivation method), ranging from 5 to 28 days (Liao et al., 2001; Islam et al., 2003; Bahrami et al., 2013; Kamiab et al., 2017; Skutnik et al., 2021), and it is also affected by pre-harvest and post-harvest conditions. The senescence of cut flowers resulted from the depletion of organic reserve compounds by respiration (Finger et al., 2016), the blocking of water uptake by air embolism (Van Doorn, 1990; Verdonk et al., 2023), xylem vascular occlusion by infection of bacteria and fungi, withering by excessive water loss, mechanical damage and presence of contaminants, such as bacteria, fungi, or the high content of salts (Da Costa et al., 2021).

The potential vase life of cut flowers is one of the most important quality factors, strongly influencing the choice of consumers and the value of cut flowers (Onozaki et al., 2001). The simplest approach to prolonging vase life involves breeding new cultivars with genetically extended post-harvest longevity (Onozaki et al., 2001). A recent study showed that “Blue Picote” (BP) and “Kroma White” (KW) are genotypes of lisianthus that have longer expected vase life than “Arena Green” (AG) and “Corelli Pink” (CP) (Kwon et al., 2024). However, consumers are attracted to the visible color and shape of cut flowers in the market, rather than unpredictable vase life. Since consumers have various preferences, breeders should be able to prolong the vase life of any cultivar via environment control and management. Although breeding has led to a spectacular cultivar in phenotypes, improving post-harvest longevity is still a challenge (Verdonk et al., 2023). Among various cultivation methods, hydroponics is gaining attention as a soil-less farming system that can produce plant materials of uniform quality while avoiding nutrient deficiencies (Ujala et al., 2024), and it is a widely used cultivation method for improving the quality of rose and lisianthus in South Korea. Different cultivation methods induce various environmental conditions such as soil, nutrient supply, and irrigation, and these factors affect the quality of cut flowers. It was shown that hydroponics results in longer average vase life of lisianthus than soil cultivation (Kwon et al., 2024).

In addition to the genotype and environment, chemical compounds have been used during the pre-harvest or post-harvest period, and chemical-based management can prolong the vase life as well. After harvest, preservative solutions containing a combination of carbohydrates (usually sugar), plant growth regulators, ethylene inhibitors, germicides, mineral salts, and organic acids have been used to increase the vase life of cut flowers. Most preservatives have been applied as post-harvest treatments, while plant growth regulators have been used before and after harvest of cut flowers. Cytokinin and gibberellins have been widely used as a component of conservative solutions in cut flowers, but it was reported that ethylene reduced their longevity and induced petal wilting, senescence and abscission (Da Costa et al., 2021). Therefore, as an ethylene inhibitor, 1-MCP has been widely used to increase the vase life. Salicylic acid (SA) is known to inhibit the production of ethylene by preventing 1-aminocyclopropane-1-carboxylate (ACC) oxidase enzyme activity in ethylene biosynthesis (Huang et al., 1993). It was also reported that SA regulates plant growth and development in response to environmental stresses (Fragnière et al., 2011) and photosynthetic rate and stomatal conductance (Chen et al., 2020). Furthermore, it has been shown that SA extends vase life by delaying senescence in cut flowers (Ravanbakhsh et al., 2017; Shabanian et al., 2019; Hajizadeh et al., 2024; Kwon et al., 2024).

A recent paper also discussed the effect of SA treatment, but the relationship between SA concentration and vase life was not consistent across the genotypes and controlled environments (Kwon et al., 2024). In that study, it was also found that petal colors measured by a colorimeter and a chlorophyll meter were strong predictors for the vase life of lisianthus cut flowers. In this study, we investigate the effects of genotype, environment, and management, and we also identify phenotypes (measured in vegetative and reproductive periods) significantly related to the vase life (measured in the post-harvest period). The data are analyzed extensively using the additive main effects and multiplicative interaction model (also known as AMMI model) and linear mixed-effects regression to better understand the genotype × environment × management interaction and key vegetative and reproductive characteristics related to the vase life of lisianthus cut flowers.

## 2 Materials and methods

### 2.1 Plant materials (genotype)

Sixty-five-day-old seedlings of four lisianthus cultivars, “Arena Green” (AG), “Blue Picote” (BP), “Corelli Pink” (CP), and “Kroma White” (KW), were planted on June 22, 2022 in the greenhouse of the Pocheon Agricultural Technology Center. The cultivation methods and environmental conditions have been described in detail in the previous study (Kwon et al., 2024).

### 2.2 Cultivation method (environment) and SA concentrations (management)

There were two experimental factors designed for the cultivation method (environment). The first factor was soil cultivation (S) or hydroponic cultivation (H), and the second factor was whether SA application was treated in the pre-harvest vegetative stage (V) or reproductive stage (R). The V stage is defined as three weeks after lisianthus seedlings develop roots in stages 1, 2, and 3 after planting. The R stage is defined as the period of SA treatment starting with development of the flower bud, when over 60–70% of flower budding has occurred in the average plant.

Four environmental conditions were designed by crossing the two factors: soil cultivation with SA treatment at vegetative stage (SSV), soil cultivation with SA treatment at reproductive stage (SSR), hydroponics with SA treatment at vegetative stage (HSV), and hydroponics with SA treatment at reproductive stage (HSR). In addition to the environmental conditions, we also investigated the effect of SA concentration at four levels (0, 0.1, 0.3, and 0.5 mM) on the vase life of cut lisianthus. The SA (Gooworl Co., Daegu, Korea) was dissolved in methyl alcohol as a 100 fold concentrate and then diluted with water for application. The SA was applied to six samples in each treatment group. Each concentration was applied three times at 3 days intervals, dispensing 10 mL each time. The control group was irrigated with 10 mL of diluted water-methyl alcohol solution.

In summary, the four conditions (environment: SSV, SSR, HSV, and HSR) and four levels of SA concentrations (management: 0, 0.1, 0.3, and 0.5 mM) were applied to four cultivars of lisianthus cut flowers (genotypes: AG, BP, CP, and KW).

### 2.3 Investigation of vegetative and reproductive characteristics

The vegetative characteristics measured in this study were stem diameter (mm), stem node (no.), stem length (mm), stem bush (no.), flowering day (day), and SPAD (value). Stem diameter was measured in terms of the growth after planting at the fourth or fifth node from the bottom.

To investigate reproductive characteristics, the flowers were cut on August 31, 2022 for the S cultivation groups (Day 68) and September 16, 2022 for the H cultivation groups (Day 83). Considering factors such as flower size and flower opening (whether the flower was 60–70% open), we selected and cut flowers that had bloomed a similar amount. The measured reproductive characteristics were fresh weight (mg), dry weight (mg), weight difference (mg), petal number (no.), petal size (mm), and vase life (day). Lisianthus cultivars show differences in their flowers, stems, and the appearance and location of their leaves; hence, the flower stems were cut 5 cm long to create equal conditions. All lower leaves were removed. The fresh weight was measured as the biomass on the day of measurement, and the dry weight was measured in the dry state at 30 days after flower cutting. The weight difference is a derived variable calculated by subtracting the dry weight from the fresh weight. The vase life was measured in a laboratory at a constant temperature of 20.4°C, relative humidity of 60–76%, and illumination of 7.43–9.45 µmol m^2^ s^-1^. The vase life of each sample was defined as the time when an investigator observed a change of petal color and collapse of petal shape. The petal number and petal size were examined on the first day of flower cutting.

### 2.4 Leaf chemical analysis of cut lisianthus

Leaves were collected on the day of cutting to measure the concentrations of inorganic compounds including nitrogen (N), phosphorus (P), potassium (K), magnesium (Mg), and calcium (Ca) accumulated in them. After completely drying 0.5 g of leaves from the cut plants for 8 hours at 70°C, they were homogenized using a miniature homogenizer. The sample (three replicates) was digested in the 10 ml reagent mixture (DW 1,000 ml + 1,800 ml of 50% perchloric acid + 200 ml of 30% sulfuric acid) by heating block at 25°C for 8 hours and 200°C for 8 hours. After the solution cooled under 60°C, an aliquot was diluted in 100 ml mess flasks with DW and filtered by a filter paper (Whatman No. 6). Total N was analyzed using the Kjeldahl method (Bradstreet, 1954), P was analyzed using the Vanadate method (Cavell, 1955), and K, Mg, and Ca were analyzed using Inductively Coupled Plasma–Optical Emission Spectrometry (Integra XL ICP-OES, GBC Scientific Equipment, Victoria, Australia). The concentrations of all chemicals were measured in mg g^-1^ of dry weight.

### 2.5 Statistical analysis

Given the four genotypes (AG, BP, CP, and KW) and four environments (HSR, HSV, SSR, and SSV), the AMMI model was implemented to test for the genotype × environment interaction (GEI), and the biplot was used to graphically represent the GEI. The mixed-effects model was used to estimate the mean vase life, and a 95% confidence interval (CI) was calculated for each level of GEI.

Given the four levels of management (SA concentrations of 0, 0.1, 0.3, and 0.5 mM), the ANOVA was performed to test whether or not the additive effect of management exists given the GEI under the mixed-effects model. This model assumes that the effect of management is constant across all levels of GEI, and an additional ANOVA was performed to further test whether or not the genotype × environment × management interaction (GEMI) exists under the mixed-effects model. The models were used to estimate the mean vase life and calculate a 95% CI for each level of GEI and of GEMI.

As secondary analyses, given GEMI under the model, we investigated which of the vegetative characteristics (stem diameter, stem nodes, stem length, stem bush, flowering day, and SPAD), reproductive characteristics (fresh weight, dry weight, petal number, and petal size), and chemical components (N, P, K, Mg, and Ca) are related to the vase life. We identified significant vegetative characteristics, reproductive characteristics, and chemical components separately, and then combined all significant characteristics in the mixed-effects model to account for potential confounding variables. After identifying significant variables, the response-surface method (RSM) was used to identify the best combination of factors associated with the vase life.

All statistical analyses were performed in R with the following packages: metan, lme4, lmerTest, and rsm (Bates et al., 2015; Kuznetsova et al., 2017; Lenth, 2009; Olivoto and Lúcio, 2020).

## 3 Results

**Figure 1:**
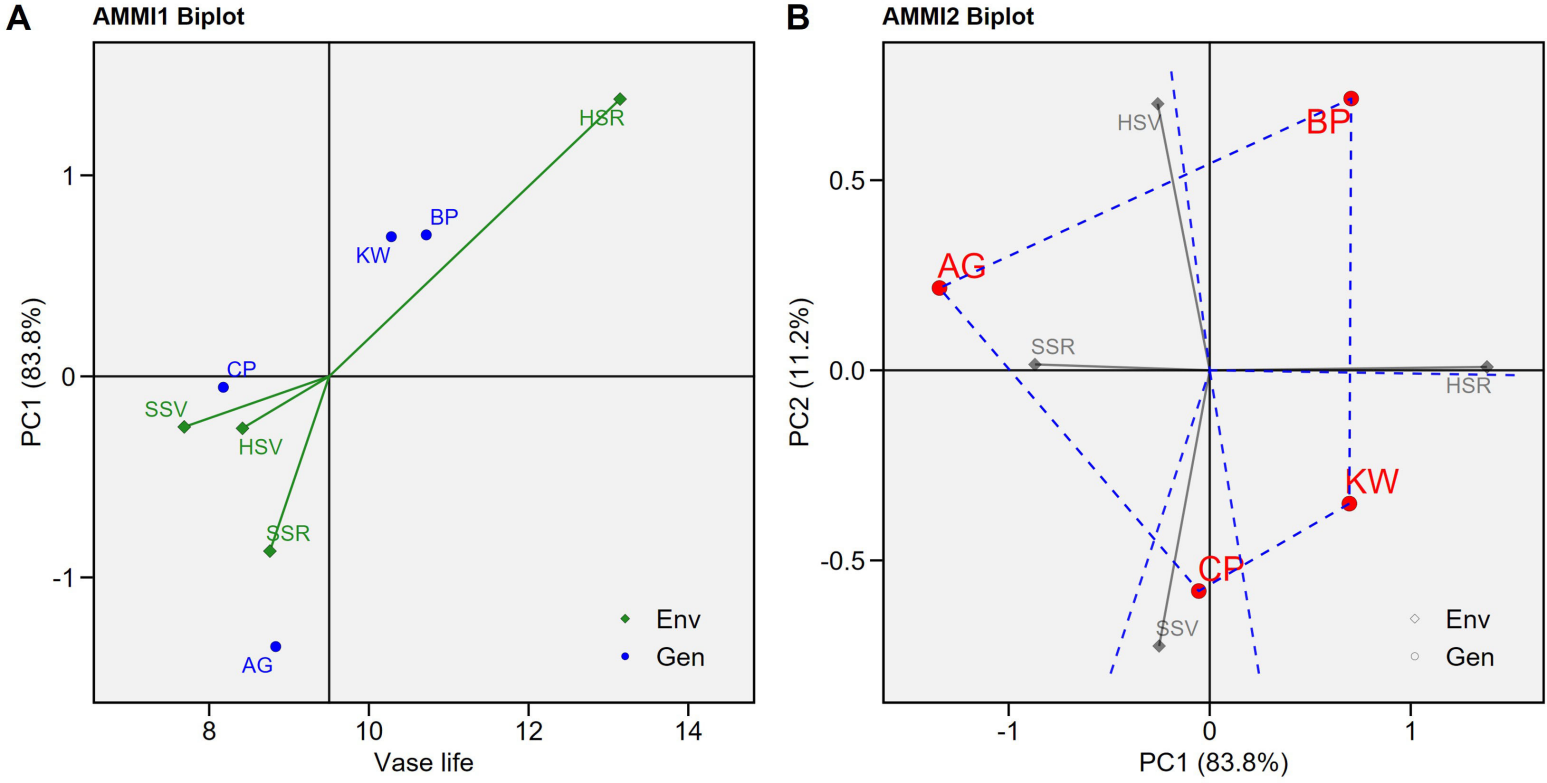
The AMMI1 **(A)** and AMMI2 **(B)** biplots for vase life (days) of the four genotypes (Arena Green (AP), Blue Picote (BP), Corelli Pink (CP), and Kroma White (KW)) under the four environments (HSR: hydroponics with salicylic acid (SA) treatment at reproductive stage), HSV: hydroponics with SA treatment at vegetative stage, SSR: soil cultivation with SA treatment at reproductive stage, and SSV: soil cultivation with SA treatment at vegetative stage).

Under the AMMI model, the GEI was significant (p < 0.001). **Figure 1A** shows the biplot of the four genotypes (AG, BP, CP, and KW) in the four environments (HSR, HSV, SSR, and SSV). In the AMMI1 biplot, the biplot abscissa (x-axis) indicates the target variable, vase life, and the ordinate (y-axis) indicates the first principal component (PC1). This plot shows the variation of the main additive effect of environment and genotype on the x-axis, and the variation of the multiplicative effect of GEI on the y-axis (Oroian et al., 2023). The genotypes and environments located on the right side of the vertical line have higher average vase life values than those on the left side (Hossain et al., 2023). BP had the highest average vase life (10.72 days) followed by KW (10.28 days). These genotypes had greater average vase life than the overall average indicated by the vertical line of the AMMI1 biplot. The lowest average vase life was observed by CP (8.18 days) followed by AG (8.83 days). The only environment that has a greater average vase life than the overall average is HSR (13.15 days), and the biplot demonstrates that hydroponic cultivation with SA treatment during the reproductive period is the most suitable environment to prolong the vase life of cut lisianthus.

If a genotype or environment has a PC1 score that is near zero, it implies a minimal interaction effect (Hossain et al., 2023). CP reacted to HSR significantly like the other genotypes, but it was not affected by the other treatments (to be shown in the subsequent figure). On the other hand, the effects of the four environments on the vase life of AG were quite different when compared to the other three genotypes, so AG has the largest absolute PC1 score in the AMMI1 biplot. The biplot also shows that the variability of vase life due to the environment is substantially larger than due to the genotype, and resulting statistics of the AMMI model support it (**Table 1**). About 35% of the variation of vase life was due to the environment, 8% of the variation was due to the genotype, and an additional 4% was due to the GEI. **Figure 1B** shows the additive main effect and multiplicative interaction (AMMI2 biplot) using PC1 and PC2 (Oroian et al., 2023), and the first two PCs explained 94.9% of the variation of vase life (83.9% and 11%, respectively). The HSR environment is located at the positive side of the x-axis (PC1), and the SSR environment is located at the negative side of the x-axis. These opposite sides imply that hydroponics cultivation and soil cultivation had substantially different effects even though SA was treated during the same reproductive period. The HSV environment is located at the positive side of the y-axis (PC2), and the SSV environment is located at the negative side of the y-axis. These two environments also had different effects even though SA was treated during the same pre-harvest vegetative period. The AMMI2 biplot shows that, given the magnitudes of separation, the effects of HSR and SSR are more different than the effects of HSV and SSV.

**Table 1:** ANOVA for the vase life (AMMI model).

Source	DF	SS (% Total)	MS	F-value	P-value
Environment (E)	3	1749.95 (34.99%)	583.32	19.85	<0.001
Block	12	352.71 (7.05%)	29.39	4.70	<0.001
Genotype (G)	3	417.07 (8.34%)	139.02	22.21	<0.001
E × G interaction	9	221.93 (4.44%)	24.66	3.94	<0.001
PC1	5	31.07 (0.62%)	6.21	0.99	0.424
PC2	3	4.09 (0.08%)	1.36	0.22	0.883
PC3	1	1.89 (0.04%)	1.89	0.30	0.584
Residuals	355	2222.08 (44.43%)	6.26		
Total	391	5000.78 (100.0%)	12.79		

**Figure 2:**
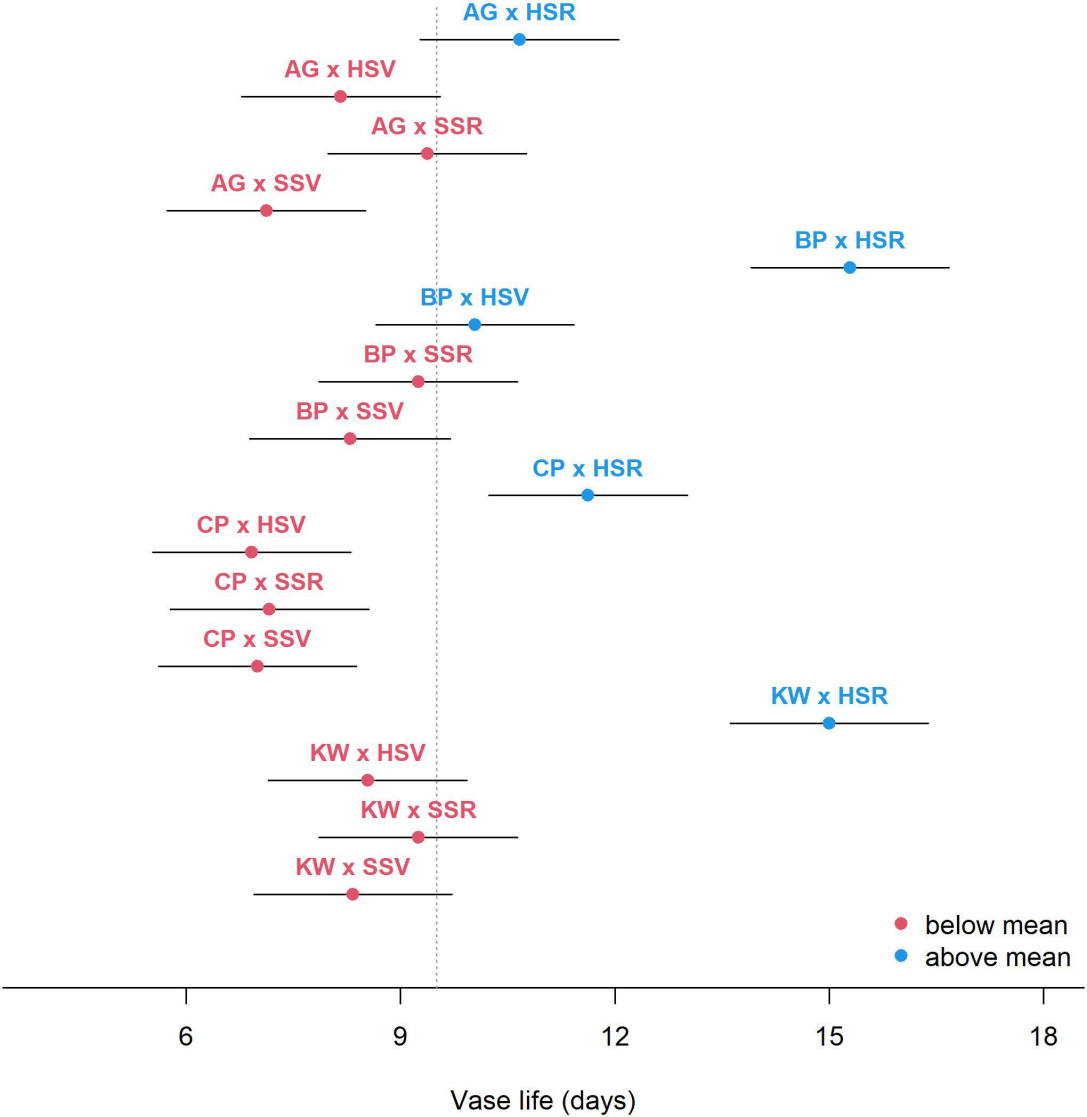
Estimated mean vase life (circle) and 95% CI (bar) for each combination of genotype × environment. The vertical dashed line shows the average of the vase life of all combinations.

**Figure 2** shows the estimated mean vase life with a 95% CI for each level of GEI. In general, the HSR environment prolonged the vase life, and the HSR effect was especially stronger for BP, CP, and KW, but not as strong for AG. Though the estimated mean vase life was similar between AG × HSR and CP × HSR, the effect of HSR was quite substantial for CP when compared to the other environments within each genotype. According to the mixed-effects model estimates, the mean vase life was the highest for BP with the HSR environment (15.29 days) followed by KW × HSR (15.00 days), and CP × SSV resulted in the shortest mean vase life (8.54 days).

**Figure 3:**
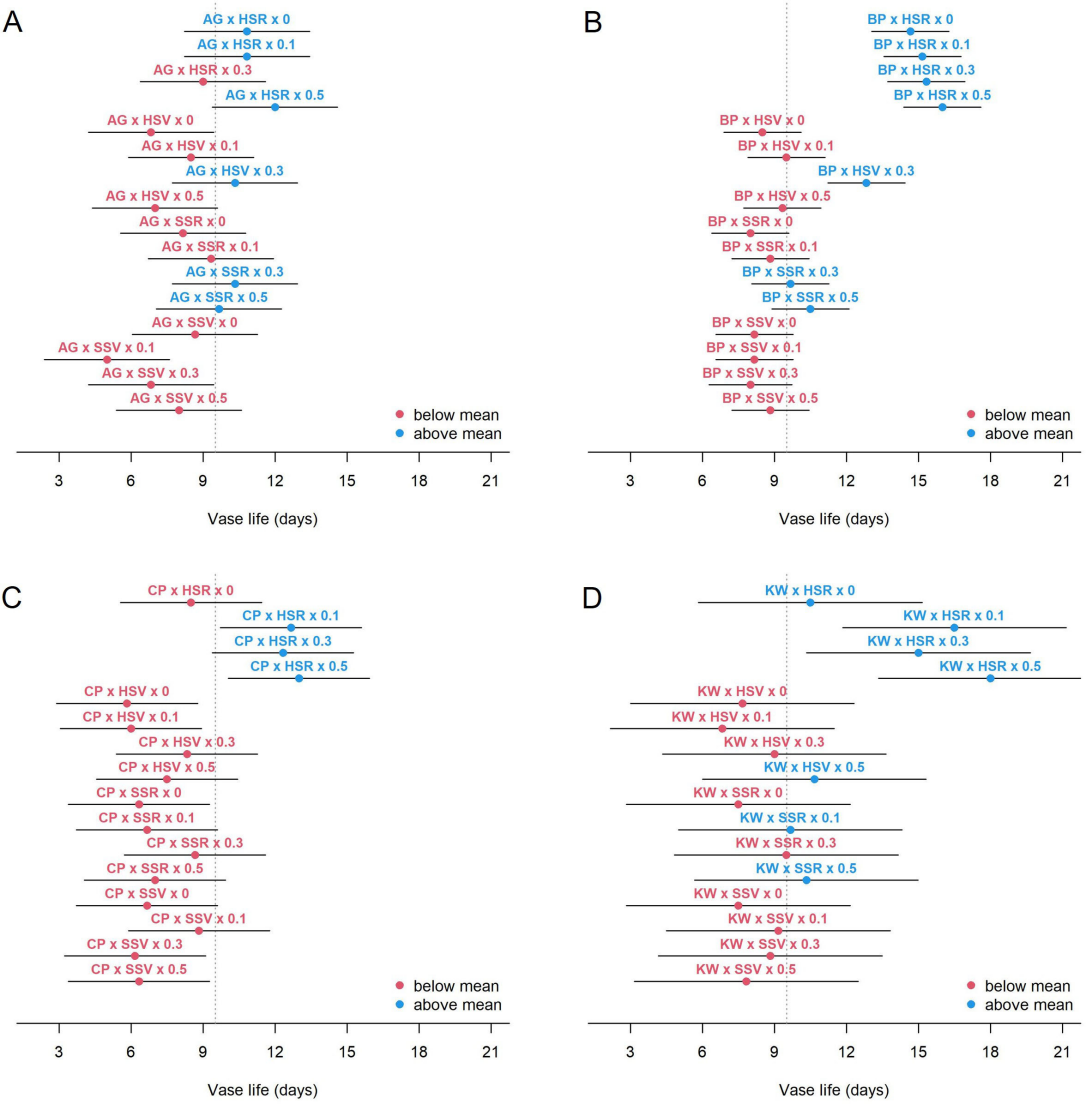
Estimated mean vase life (circle) and 95% CI (bar) for each combination of genotype × environment × management for AG **(A)**, BP **(B)**, CP **(C)**, and KW **(D)**. The vertical dashed line shows the average of the vase life of all combinations.

The additive effect of management (SA concentration) was significant given GEI (p = 0.010). When compared to the control concentration (0 mM), given GEI, the expected vase life increased by 1.08, 1.62, and 1.73 days at the SA concentration of 0.1, 0.3, and 0.5 mM, respectively. These estimates assume that the management effect is constant across all levels of GEI, and more detailed results of GEMI are shown in **Figure 3**. The figure presents the estimated mean vase life and 95% CI for all levels of GEMI. It shows that the mean vase life of BP was strongly influenced by the HSR environment, but the effect of SA management was rather minimal with BP × HSR (the upper right panel of **Figure 3**). On the other hand, the effect of SA management was substantial for CP × HSR (the lower left panel) and for KW × HSR (the lower right panel), and the GEMI effect was significant (p < 0.001). In other words, the management effect is specific to genotype and environment.

**Figure 4:**
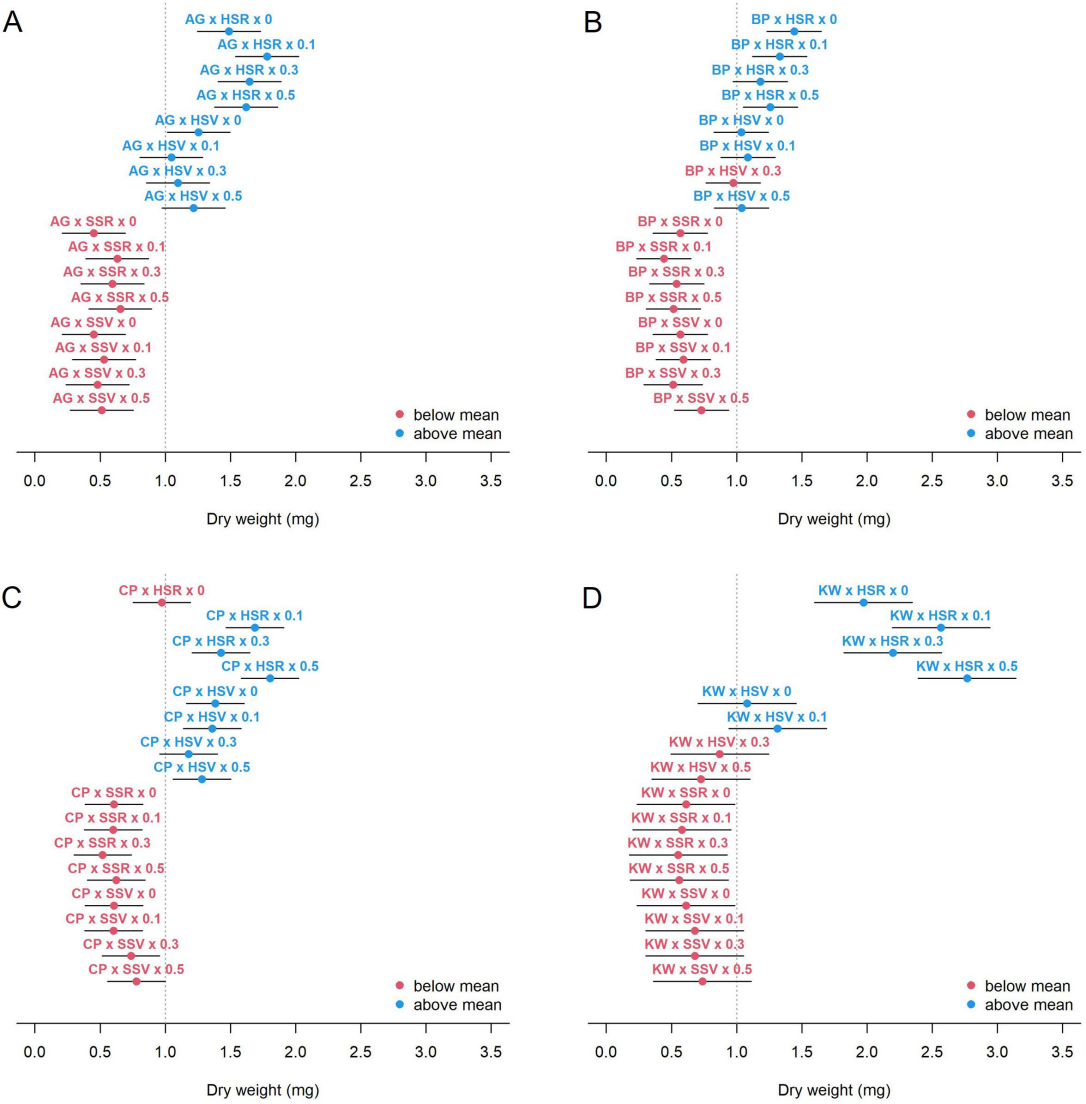
Estimated mean dry weight (circle) and 95% CI (bar) for each combination of genotype × environment × management for AG **(A)**, BP **(B)**, CP **(C)**, and KW **(D)**. The vertical dashed line shows the average of the dry weight of all combinations.

Under the influence of GEMI, the dry weight showed a similar trend, but a clearer pattern, when compared to the vase life (**Figure 4**). All cultivars increased dry weight under the hydroponics (H) environment, but the variation of dry weight with SA concentration was cultivar-specific. BP actually decreased in dry weight with increasing SA concentration under HSR, but the other cultivars did not. CP and KW showed a tendency of increasing dry weight with respect to SA concentration, but showed a concentration-specific response and decreased dry weight at 0.3 mM. There was a significant difference in the mean dry weight of BP and KW, which have relatively long vase life. The dry weight of KW was significantly increased by HSR environment and SA treatment, suggesting that its biomass serves as a carbohydrate source to maintain a long vase life. However, unlike KW, the dry weight of BP was smaller than that of AG or CP, suggesting that BP may have other pathways, such as smooth water uptake or resistance to vascular occlusion or withering, rather than the role of carbohydrate source to maintain its vase life.

**Table 2** summarizes the results from the mixed-effects model for the relationship between the vase life and each characteristic given the GEI interaction. The stem nodes and flowering day were identified as significant vegetative characteristics. Holding all other vegetative characteristics constant, the expected vase life was shorter by 0.53 days per stem node (p = 0.004), and it was longer by 0.06 days per flowering day (p = 0.022). All of the reproductive characteristics were significant, including the interaction between petal number and petal size. Holding all other reproductive characteristics constant, the expected vase life was 0.73 days longer per 1 mg of fresh weight (p = 0.002), and it was 1.30 days longer per 1 mg of dry weight (p < 0.001). The relationship between the vase life, petal number, and petal size (the interaction) will be described later in this section. Potassium was the only chemical component which was significant. Holding all other chemical components constant, the expected vase life was 2.18 days shorter per 1 mg g-1 of potassium content (p = 0.040).

**Table 2:** Estimated regression parameters for the vegetative and reproductive characteristics and chemical components under the linear mixed-effects regression.

Characteristic	Estimate	SEter	T-value	P-value
Stem diameter	0.019	0.286	0.067	0.946
Stem nodes	-0.534	0.179	-2.99	0.004^**^
Stem length	0.026	0.022	1.171	0.243
Stem bush	-0.163	0.162	-1.008	0.314
Flowering day	0.062	0.027	2.309	0.022^*^
SPAD	0.011	0.008	1.241	0.216
Fresh weight	0.731	0.237	3.083	0.002^**^
Dry weight	1.298	0.378	3.43	<0.001^***^
Petal number	2.244	0.591	3.8	<0.001^***^
Petal size	0.408	0.151	2.708	0.007^**^
Petals × Petal size	-0.044	0.013	-3.521	<0.001^***^
Nitrogen	0.056	1.128	0.05	0.961
Phosphorus	0.427	0.483	0.884	0.381
Potassium	-2.183	1.025	-2.13	0.040^*^
Calcium	-0.632	6.473	-0.098	0.922
Magnesium	2.377	3.494	0.68	0.499

**Table 3** summarizes all significant characteristics considered simultaneously. All of the reproductive characteristics (fresh weight, dry weight, petal number, petal size, and petal number × petal size interaction) were shown to be significant when the three kinds of characteristics were considered together. One interesting result is that the estimated interaction parameter (petal number × petal size) was negative and statistically significant (p < 0.001). That is, the positive relationship between petal size and vase life can be weakened or even reversed when a cut flower has a large petal number.

**Table 3:** Estimated regression parameters when all significant characteristics in **Table 1** are included in the linear mixed-effects regression.

Characteristic	Estimate	SE	T-value	P-value
Stem nodes	-0.354	0.173	-2.048	0.042^*^
Flowering day	0.051	0.024	2.079	0.038^*^
Dry weight	1.277	0.372	3.428	<0.001^***^
Fresh weight	0.698	0.235	2.967	0.003^**^
Petal number	2.27	0.585	3.878	<0.001^***^
Petal size	0.418	0.149	2.804	0.005^**^
Petals × Petal size	-0.045	0.012	-3.603	<0.001^***^
Potassium	-0.866	0.694	-1.248	0.220

Significance is indicated by * (p < 0.05), ** (p < 0.01), and *** (p < 0.001).

**Figure 5:**
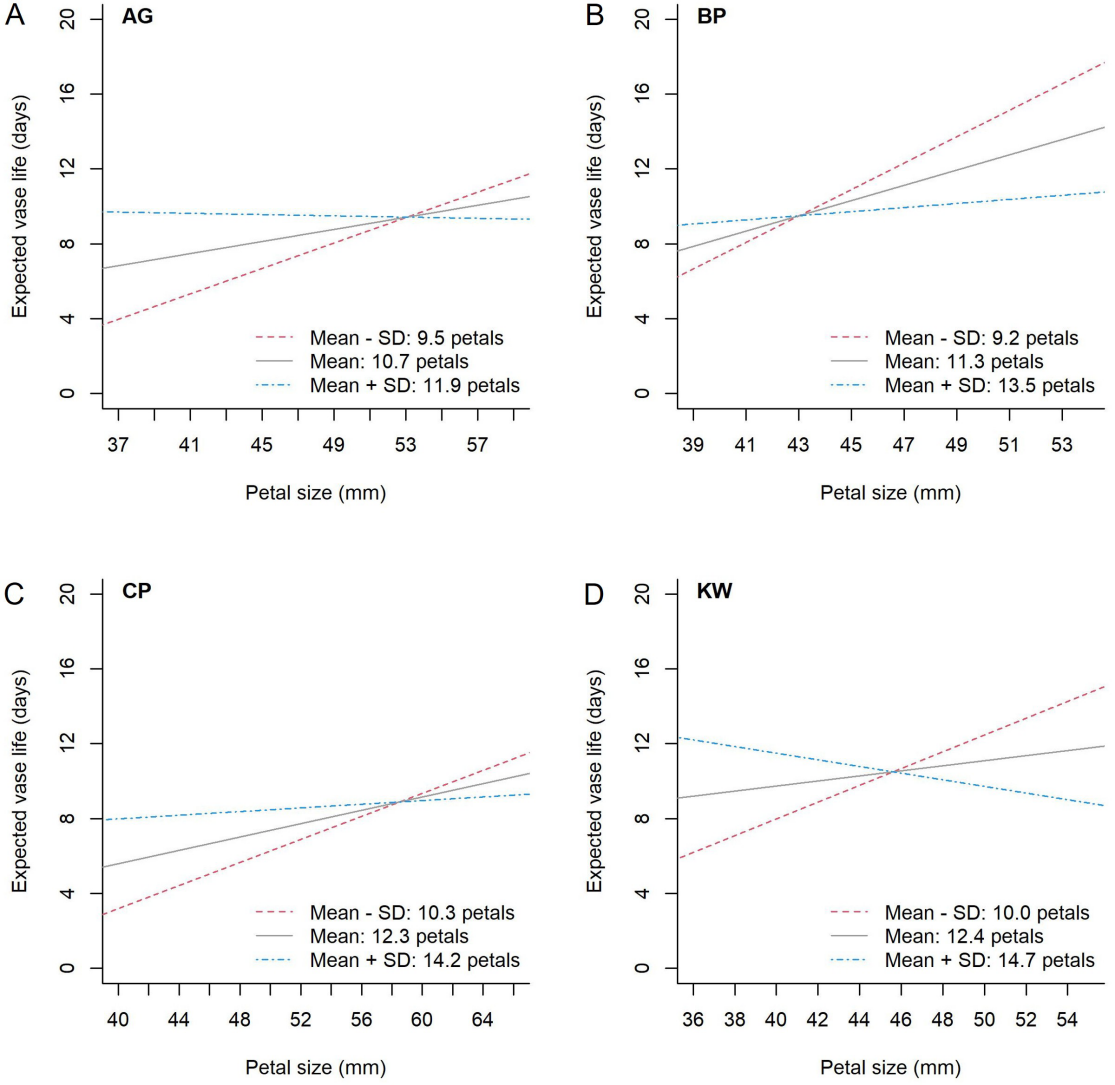
The estimated relationship between petal size and expected vase life by petal number for each genotype, AG **(A)**, BP **(B)**, CP **(C)**, and KW **(D)**. The difference in standard deviation from the mean petal number (black solid line) for each genotype is shown as a blue dash-single dotted line for greater than, and a red dotted line for less than.

To further investigate whether the interaction between petal size and petal number is significant or not for each genotype, the model was fitted for each cultivar, and the results are demonstrated in **Figure 5**. For instance of KW (**Figure 5D**), the expected vase life increases with respect to the petal size at the mean petal number (12.4 petals) or one SD below the mean (10.0 petals), but it decreases at one SD above the mean (14.7 petals). For the other genotypes (AG, BP, and CP), the expected vase life is nearly constant at one SD above the mean petal number, and it increases at the mean or lower petal number. This interaction was statistically significant for each genotype (p = 0.002, 0.010, 0.048, and 0.0005 for AG, BP, CP, and KW, respectively).

**Figure 6:**
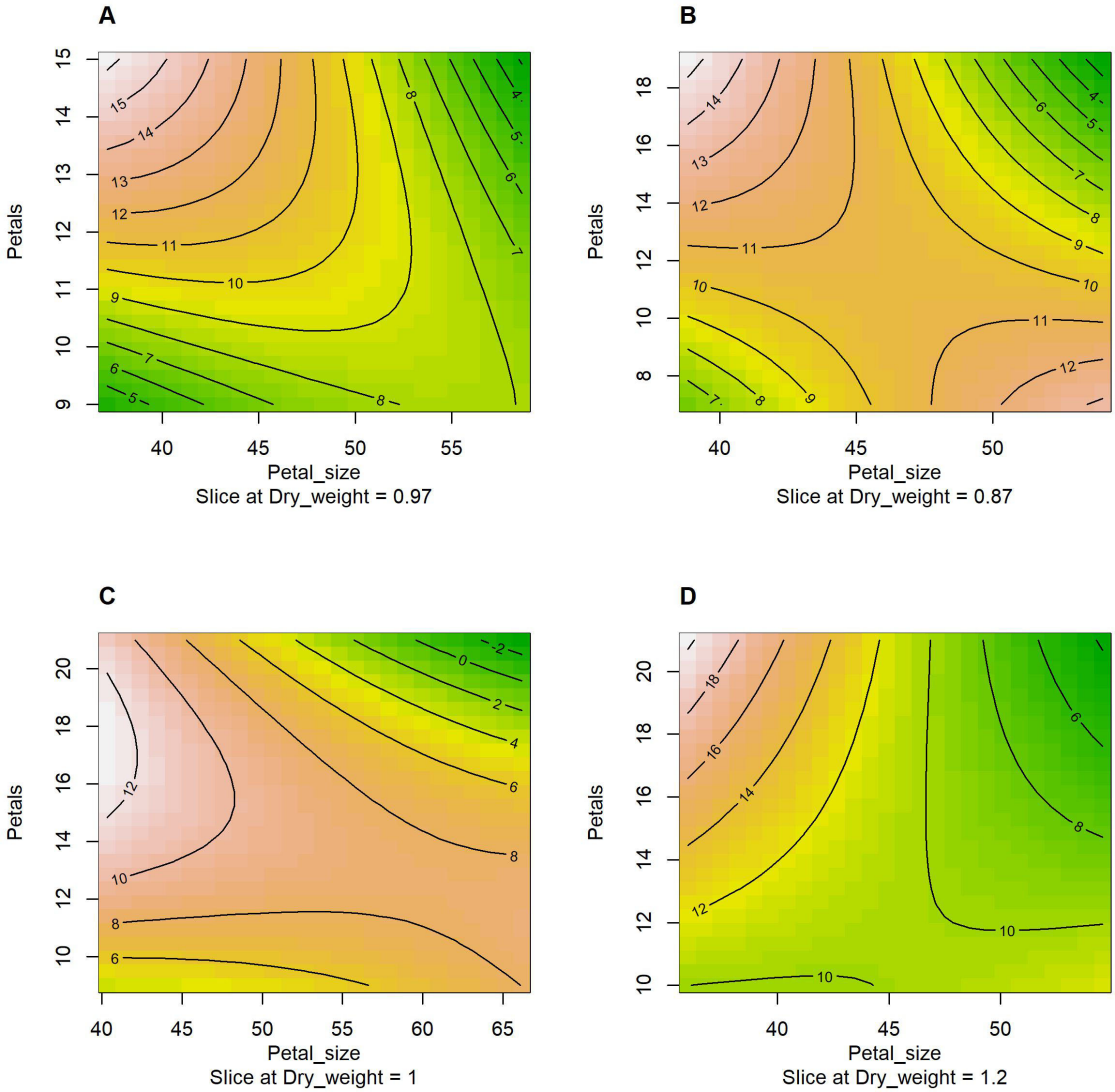
The contour plots generated by the response-surface method with the first order interaction and the second order of petal size (x-axis) and petal number (y-axis) given the dry weight and vase life. The contour lines indicate vase life sliced at the sample mean value of dry weight and the reference level of environment × management (HSR × 0 mM). **(A)** AG; **(B)** BP; **(C)** CP; and **(D)** KW.

**Figure 6** provides the estimated response-surface of vase life using the RSM analysis for each genotype. The model included the environment and management factors, the dry weight, the first-order interaction between petal number and petal size, and the second order of petal number and petal size, and it estimated that a small petal size and a high petal number maximizes the expected vase life for all four genotypes. On the other hand, a large petal size and a high petal number is not a positive sign for the expected vase life. This result supports that an adequate petal number entails well-developed tissues responsible for water supply (xylem vessels and vascular bundles), and it is essential for prolonged vase life of cut flowers (Guo et al., 2023). According to the pattern observed in **Figures 5** and **6**, it appears that having both a large petal number and a large petal size is not optimal, and having both a small petal number and a small petal size is not optimal either. The results inform the balance between the two characteristics is crucial for each cultivar.

## 4 Discussion

BP has the smallest CI, meaning there is not much variation between individuals in vase life (**Figure 3B**). Under all environments and all management, the mean vase life of BP is relatively longer than other cultivars. In other words, BP has a genome with a long vase life. Like the other cultivars, BP showed a significant increase in vase life in response to the HSR environment, and vase life tended to increase with increasing SA concentration, but the difference was not statistically significant. KW is the cultivar with the longest CI, and its vase life is strongly influenced not only by genotype, but also by environment and management (**Figure 3D**). KW was estimated to have a longer vase life than BP under HSR environment and SA concentration of 0.5 mM. Like BP, KW also has a longer vase life than the total average vase life under HSR and SA concentration control (0 mM), but the trend of increasing vase life with increasing SA concentration was different from that of BP. KW showed statistically significant differences in vase life as SA concentration increased.KW appears to have the potential to have a longer vase life than BP under certain environments and certain management. On the other hand, AG is limited in its ability to promote vase life, despite being influenced by the environment and management (**Figure 3A**). This is likely due to the genetic characteristic that AG has a short vase life. CP was particularly responsive to the HSR environment and the increase in SA concentration (management). Like KW, vase life increased with increasing SA concentration in CP, but the average vase life of HSR × SA concentration control (0 mM) was lower than the total average, indicating that vase life was not improved in the Hydroponics (H) cultivation alone, unlike BP. That is, CP must simultaneously address the environment and management to promote vase life. CP cannot be expected to improve vase life at any SA concentration under any environment other than the HSR environment. As shown in **Figure 1A**, CP is the most genotype-influenced cultivar, which means that it is genetically stable and is not significantly affected by environment and management.

One important finding of this study is that the relationship between vase life and petal size depends on the petal number for all genotypes. **Figure 5** graphically presents the expected vase life with respect to petal size at a given petal number for each genotype. In this figure, we can find the genotype-specific intersection of petal size and expected vase life: (53.1, 9.4) for AG, (43.0, 9.5) for BP, (58.5, 8.9) for CP, and (45.6, 10.5) for KW. For example, in **Figure 5A**, the model estimates that the expected vase life of AG is 9.4 days when the petal size is 53.1 mm for any petal number. When the petal size is smaller than 53.1 mm, a higher petal number is preferred for longer expected vase life. When the petal size is greater than 53.1 mm, a lower petal number is preferred. For AG and CP (**Figures 5A, C**), the slopes of the intersecting point on the blue line (when the petal number is the highest) converge to zero, showing that there is a limit to the improvement of vase life. In other words, these two cultivars have a genetic limit to vase life at higher numbers of petals. KW is longer than BP with an expected vase life of 10.5 days at a crossing point of 45.6 mm (**Figures 5B, D**). KW has the potential to have a longer vase life than BP due to the influence of GEMI. KW has the largest difference in slopes of the three curves at the contact point. BP has a positive slope for all curves (regardless of the petal number), so the difference in vase life for each individual BP cultivar is small. However, for KW, the slope of the blue line (the highest petal number) is negative. This means that the variation in vase life increases with the petal number and petal size, which lead to a large difference in vase life among KW individuals (**Figure 4D**).We can better understand the interaction between petal number and petal size using a mathematical representation as follows. When we model the genotype-specific vase life by the standardized petal number (X) and petal size (Z) and their interaction (XZ), in other words β_0_ + β_1_ X + β_2_ Z + β_3_ XZ, the overall mean vase life can be expressed as μ = β_0_ + β_3_ ρ, where ρ is the correlation coefficient between petal number and petal size. In this expression, β_0_ is the mean vase life at the mean petal number and mean petal size, and we can see that the mean vase life depends on ρ. The resulting interaction parameter is negative for all genotypes, that is β_3_ < 0, so a genotype with ρ < 0 results in a higher mean vase life due to the interaction relationship presented in **Figure 5**. BP had a negative correlation between petal number and petal size (ρ = –0.355), and it contributed to the high mean vase life. On the other hand, KW showed a weak positive correlation between petal number and petal size (ρ = +0.132), which acted against the mean vase life, and the mean vase life of KW is still as high as BP. It could imply that the environment and management positively affected KW more than BP. The dry weight was identified as another key characteristic associated with the vase life, and the effects of HSR with SA concentration were stronger on the vase life of KW than of BP as shown in **Figure 4**. In addition, we can also approximate the variance of vase life as σ^2^ = (β_1_)^2^ + (β_2_)^2^ + (β_3_)^2^ (1 – ρ^2^) + 2β_1_ β_2_ ρ under mild assumptions. According to this expression, a strong degree of interaction (i.e., large magnitude of β_3_) with a weak correlation between petal number of petal size (i.e., small magnitude of ρ) is a source of variability of the vase life, and it partially explains why the vase life of KW (|β_3_| = 1.318, |ρ| = 0.132) is more variable than of BP (|β_3_| = 1.005, |ρ| = 0.355). As reported in **Table 4**, the model-based estimates for μ and σ are very close to the calculated sample mean and standard deviation of vase life from the data.

**Table 4:** Estimated regression parameters of the interaction model (β_0_, β_1_, β_2_, and β_3_), model-based estimates for the mean and standard deviation of vase life (μ and σ) using the interaction relationship, and calculated sample mean and sample standard deviation of vase life (mean and std).

Cultivar	β_0_	β_1_	β_2_	β_3_	ρ	μ	mean	σ	std
AG	8.799	0.694	0.882	-0.974	-0.036	8.834	8.833	3.355	3.423
BP	10.395	-0.654	1.374	-1.005	-0.355	10.751	10.747	3.391	3.336
CP	8.012	0.638	0.811	-0.589	-0.283	8.179	8.177	2.945	2.970
KW	10.454	0.105	0.570	-1.318	0.132	10.279	10.281	4.071	4.039

The RSM analysis allows us to estimate and graphically show (contour plot) the expected vase life due to the interaction between petal number and petal size given the environment and management factors, the dry weight, and the second order of petal number and petal size. **Figure 6** shows that a high petal number and a small petal size is a common sign of long expected vase life for all four genotypes (AP, BP, CP, and KW). For BP a low petal number and a large petal size is another sign of long expected vase life as shown in **Figure 6B**. In addition, the RSM showed that having a high petal number and a large petal size is a common sign of short expected vase life for all four genotypes.

Optimal conditions (environment, management, dry weight, petal size, and petal numbers), which yield the maximal expected vase life for each cultivar, can be estimated by the RSM and grid search. Under the RSM model within the observed range of each condition, the optimal conditions for AG are SSR × 0.3 mM, a dry weight of 3.4 mg (observed range: 0.23–3.4), a petal size of 37.2 mm (37.2–58.6), and a petal number of 15 (9–15), and the conditions are expected to yield a vase life of 27.2 days (3–19). The optimal conditions for BP are HSR × 0.5 mM, a dry weight of 2.2 mg (observed range: 0.3–2.2), a petal size of 38.8 mm (38.8–54.0), and a petal number of 19 (7–19), and the conditions are expected to yield a vase life of 21.8 days (3–16). The optimal conditions for CP are HSR × 0.5 mM, a dry weight of 2.4 mg (observed range: 0.3–2.4), a petal size of 40.3 mm (40.3–66.2), and a petal number of 17 (9–21), and the conditions are expected to yield a vase life of 17.3 days (3–16). The optimal conditions for KW are HSR × 0.5 mM, a dry weight of 4.8 mg (observed range: 0.4–4.8), a petal size of 36.0 mm (36.0–54.6), and a petal number of 21 (10–21), and the conditions are expected to yield a vase life of 31.1 days (5–22). These results demonstrated variations in the optimal conditions, necessitating tailored cultivation protocols for maximizing the expected vase life between the four genotypes. A high petal number and small petal size are correlated with enhanced vase life, especially for AG and KW, the expected maximal vase life under the optimal conditions can be longer than the observed maximum days by 8 and 9 days, respectively.

In this study, the dry weight is a key characteristic related to the vase life. As shown in **Figure 4**, in the HSR environment, the dry weight of BP was not significantly affected by SA application, while the dry weight of AG, CP, and KW were increased by SA treatment. For example, a SA concentration of 0.1 mM appears to be sufficient for increasing the dry weight of AG, and a concentration of 0.5 mM may further increase the dry weight of CP and KW, but any nonzero concentration does not seem to be helpful for the dry weight of BP.

**Figure 7:**
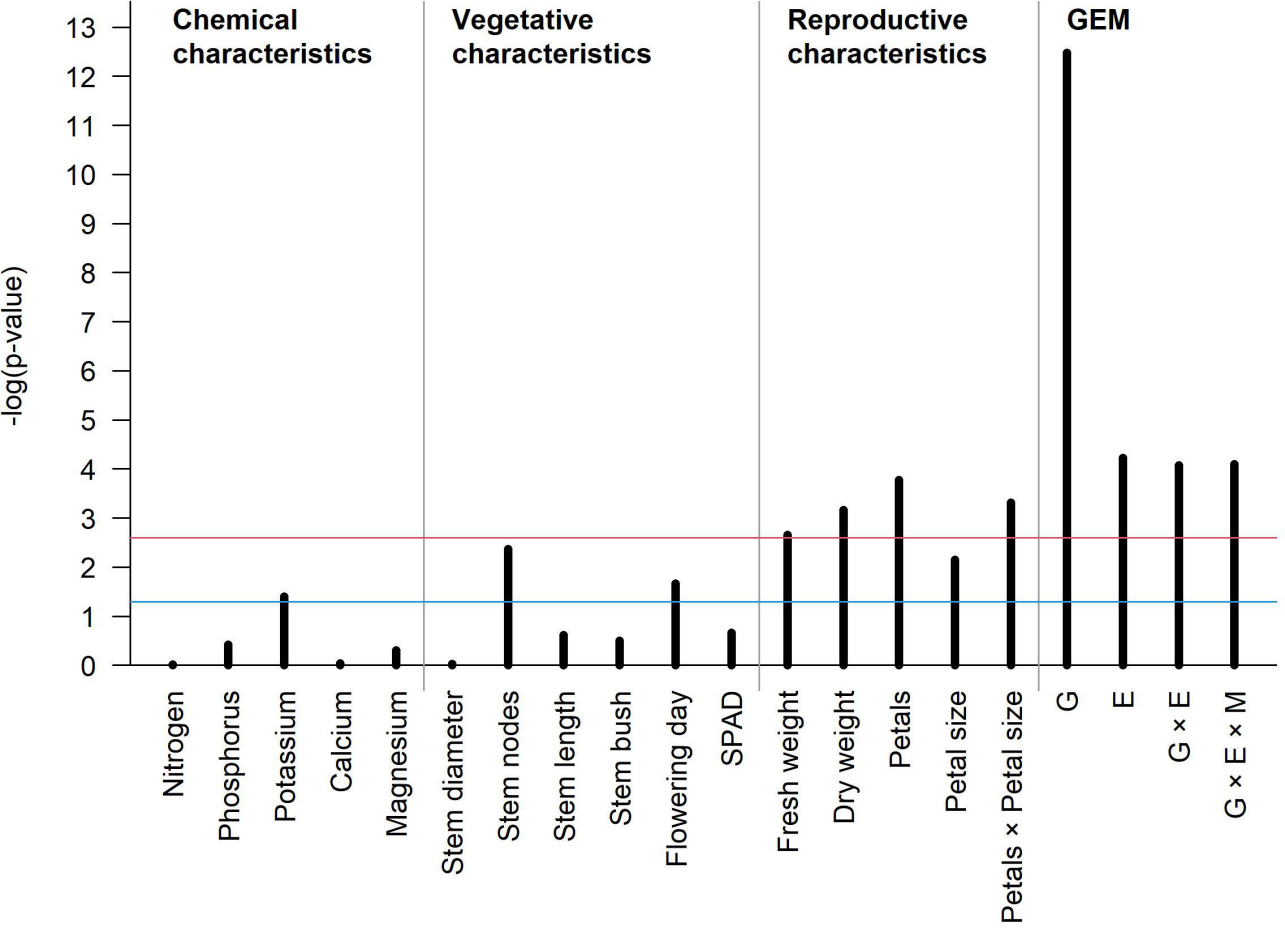
The barplot showing the significance of association between vase life and several phenotypes of cut lisianthus. The x-axis shows the different phenotypes, and the y-axis reflects the p-value for each phenotype. The blue and red horizontal lines represent an unadjusted significance level of 0.05 and a family-wise significance level of 0.05 by the Bonferroni correction, respectively.

The barplot in **Figure 7** summarizes the statistical significance of G, E, GEI, and GEMI and all characteristics under the linear mixed-effects regression model for the vase life. When we accounted for all significant variables, the R-square value of this model was 0.67, and among the various characteristics, dry weight and petal number × petal size were important parameters of this model. The vase life of cut lisianthus showed a clear difference according to genotype, and it was also found to be significantly affected by environment and GEI. Even though the effect of GEMI was significant, its magnitude was rather small when compared to the effect of G alone. Our in-depth statistical analysis suggests that farmers’ cultivar selection is the most important for the vase life, and the cultivar specific manuals for environmental control and agricultural management practice can further improve the vase life.

## 5 Conclusions

The expected vase life of cut lisianthus differs by genotype, environment, and management, and their interaction is significant. This finding suggests cultivation and management manuals specific to each cultivar. The SA treatment affected the dry weight, which is shown to be one of the most significant variables explaining the vase life under the linear mixed-effects regression model. In addition, the petal number × petal size interaction was also significant for all cultivars. The RSM analysis shows that a high petal number and a small petal size is a positive indication for the vase life of all four cultivars, whereas a high petal number and a large petal size is a negative indication for the vase life of all four cultivars, and the optimal balancing point between the two reproductive characteristics is specific to each cultivar.

## Data Availability

The raw data supporting the conclusions of this article will be made available by the authors, without undue reservation.
